# Hydrocarbon degradation and genomic insights of *Klebsiella pneumoniae* from oil-contaminated soils in Guimaras Island, Philippines

**DOI:** 10.3389/fmicb.2026.1782430

**Published:** 2026-03-18

**Authors:** Kalel Liam M. Garcia, Darlene Kris V. Alemania, Rina B. Opulencia

**Affiliations:** Institute of Biological Sciences, College of Arts and Sciences, University of the Philippines Los Baños, Laguna, Philippines

**Keywords:** bioremediation, genomics, *Klebsiella pneumoniae*, microbial hydrocarbon degradation, oil spill

## Abstract

*Klebsiella* was previously found to be the dominant genus of a hydrocarbon-degrading, diesel-enriched microbial consortium from oil-spill contaminated soils in Guimaras Island, Philippines. However, the species-level identity, individual degradation efficiency, and genomic determinants of metabolic pathways and environmental stress adaptation remain unknown, limiting the understanding of the ecological role and bioremediation potential of *Klebsiella* species. This study isolated, characterized, and evaluated the hydrocarbon degradation efficiencies of *Klebsiella* species from the consortium. From 110 putative *Klebsiella* colonies, 11 isolates showed significantly different degradation capacities of at least 66% on diesel, hexane, hexadecane, and xylene, suggesting diversity at the species and strain level, and substrate preference. Isolate KLMG-HD-125 demonstrated complete oxidative degradation of short- and mid-chain n-alkanes (C11–C25) in diesel, indicating a broad substrate range and strong petroleum degradation potential. Whole genome sequence analysis confirmed the identity of KLMG-HD-125 as *Klebsiella pneumoniae* with a genome size of 5.55 Mb and GC content of 57.2%. It harbors genes responsible for alkane and xylene degradation, and pathways to respond to oxidative, membrane, and solvent stress, indicating ecological fitness, allowing the isolate to remain metabolically active in potentially toxic petroleum-impacted environments. Specifically, the presence of *ladA* (LadA-like FMN-dependent monooxygenase), *adhP* (primary alcohol dehydrogenase), and *adh* (long-chain aldehyde dehydrogenase) indicates the pathway for terminal oxidation of alkanes. Although 11 virulence-associated genes were identified in KLMG-HD-125, the hypervirulence determinants were absent. Together with its environmental origin, these findings indicate that KLMG-HD-125 is potentially opportunistic rather than an obligate pathogen. Overall, KLMG-HD-125 is equipped with phenotypic and genomic traits essential for hydrocarbon remediation, specifically of n-alkanes, and ecological fitness to persist in petroleum-impacted environments, though its potential pathogenicity requires careful biosafety assessment prior to environmental application.

## 1 Introduction

The worst oil spill in Philippine history happened on August 11, 2006, when the tanker M/T Solar 1 sunk in Guimaras Island, spilling > 2 million liters of oil, severely impacting the island and threatening coastlines, coral reefs and mangroves, including those of nearby islands of Panay and Negros (Alea et al., 2022). Several cleaning strategies such as using booms, sorbent pads and chemical dispersants were employed (Yender and Stanzel, 2011) but it is estimated that a significant portion of the spilled oil remains uncollected or undocumented (Bacosa et al., 2025). To address these limitations, the National Oil Spill Contingency Plan recommended bioremediation as a key strategy for long-term rehabilitation of affected coastal habitats (Raymundo, 2012). However, no biological bioremediation agent has been identified for this purpose.

Rodriguez et al. (2023) recently isolated and diesel-enriched microbial consortia from oil spill affected areas in Guimaras Island and showed that these were efficient in degrading diesel, xylene, hexane and hexadecane. However, the use of microbial consortium for bioremediation is often limited in stability and robustness, mainly attributed to diverse growth conditions required, dynamic characteristics, competition, and intricate communication among different microbial species (Duncker et al., 2021; Lin et al., 2024; Nnabuife et al., 2022). In contrast, monocultures can offer simpler metabolic dynamics, better reproducibility, and controlled behavior. Furthermore, with well-understood pathway, a monoculture can be more suitable in degradation of a specific compound.

Rodriguez et al. (2023) found that *Klebsiella* was the most abundant genus in all diesel-enriched microbial consortia from oil spill-affected areas in Guimaras Island that were efficient in degrading multiple hydrocarbon substrates. However, they have not been isolated as pure cultures, and the species-level identity, individual degradation efficiency, and genomic basis of hydrocarbon metabolism and environmental stress adaptation have not been elucidated, limiting the understanding of their ecological role in contaminated environments and their potential as candidates for bioremediation.

In this study, putative *Klebsiella* isolates from a diesel-enriched microbial consortium were isolated, characterized, and evaluated for their substrate-specific hydrocarbon degradation efficiencies. One isolate exhibiting at least 66% degradation across multiple hydrocarbon substrates was selected for whole genome sequencing. GC–MS analysis was used to confirm its diesel degradation capability, while genome annotation allowed the identification of genes associated with hydrocarbon degradation pathways and environmental stress responses, allowing prediction of its metabolic pathways for specific hydrocarbon degradation. Together, these analyses provide new insight into the metabolic potential of *Klebsiella* spp. native to Guimaras oil spill sites and their possible applications in future bioremediation strategies.

## 2 Materials and methods

### 2.1 Isolation and purification of *Klebsiella* species

#### 2.1.1 Revival of consortia from glycerol stock

Hydrocarbon-degrading microbial consortium obtained from Brgy. Lucmayan, Nueva Valencia, Guimaras Island, Philippines (consortium A5; Rodriguez et al., 2023) was withdrawn from -80°C and thawed on ice (Baylis, 2006). Three hundred μL of the consortium were inoculated into 30 mL of Bushnell-Haas broth (per 1,000 mL: 0.2 g MgSO_4_, 0.02 g CaCl_2_, 1.0 g KH_2_PO_4_, 1.0 g K_2_HPO_4_, 1.0 g (NH_4_)_2_SO_4_, 0.05 g FeCl_3_) supplemented with filter-sterilized 1.5% diesel and incubated at about 25°C for 7 days.

#### 2.1.2 Isolation, purification and preservation

Ten-fold dilution series (10^–1^–10^–8^) was prepared from the revived consortium and 1 mL of each dilution was spread plated on Simmons Citrate agar with 1% inositol (SCAI), which was then incubated at 37°C for 48 h. Isolated glossy and yellow colonies were selected and purified by streaking on SCAI agar plates through three consecutive rounds, with incubation at 37°C for 24 h at each round. Purified colonies were subsequently grown on nutrient agar plates at 37°C for 24 h, then into nutrient broth and incubated under the same condition. One-half mL of the culture broth was mixed with 500 μL of 95% glycerol and stored at -80°C.

### 2.2 Hydrocarbon degradation assay

#### 2.2.1 2 ,6-DCPIP assay

A modified 2,6-dichlorophenolindophenol (DCPIP) assay of Obi et al. (2016) was performed to determine the hydrocarbon degradation capability of the *Klebsiella* isolates. Each of the isolates was pre-cultured in 5 mL nutrient broth and incubated at 37°C for 24 h. Three-tenths of a mL from the actively growing cells was inoculated into 30 mL nutrient broth and incubated at 37°C for 24 h. The cells were harvested by centrifugation at 4,000 × g for 15 min and washed thrice with 5 mL of 0.9% NaCl solution. The cell pellet was resuspended using Bushnell-Haas medium until OD_600_ reached 1.0. The test sample contained 800 μL of Bushnell-Haas medium, 80 μL of cell suspension, 50 μL of 2,6-DCPIP solution (37.5 μg/mL), and 5 μL of hydrocarbon (diesel, hexane, hexadecane or xylene). The negative control did not contain cell suspension. The solution was incubated at 37°C until the color changed from blue to colorless. This assay estimates the hydrocarbon degradation when 2,6-DCPIP turns colorless as it becomes reduced (Wani et al., 2022) by electrons from oxidation of the hydrocarbon. To determine the percent degradation, the supernatant was read at 609 nm using UV-VIS spectrophotometer (BIOBASE, China) and plotted on the following formula (Obi et al., 2016):


$$\%\mathrm{degradation}=\left(1-\frac{\mathrm{Absorbance}\,\mathrm{of}\,\mathrm{treated}\,\mathrm{sample}}{\mathrm{Absorbance}\,\mathrm{of}\,\mathrm{the}\,\mathrm{control}}\right)\times 100$$

#### 2.2.2 Statistical analysis

*F*-test Analysis of Variance and Bonferroni Pairwise Comparison Test were conducted on the % hydrocarbon degradation values of the 110 putative *Klebsiella* isolates.

### 2.3 Biochemical characterization

Isolates that exhibited ≥ 66% hydrocarbon degradation on all tested hydrocarbons were subjected to the following biochemical tests: catalase, urease, oxidase, methyl red and Voges-Proskauer tests, citrate utilization, indole and H_2_S production, and fermentation of glucose, lactose, and fructose (Merchant and Packer, 1969).

### 2.4 Gas chromatography-mass spectrometry (GC-MS)

Cells of KLMG-HD-125 with OD_600_ = 1.0 were prepared as described in section 2.2.1. Ten mL of the cell suspension was mixed in a 250 mL Erlenmeyer flask with 89 mL of Bushnell-Haas medium and 1 mL of diesel. After incubation at 35°C for 14 days, the organic phase of the solution was separated using *n*-hexane on separatory funnel. The obtained solution was filtered using a sterile 0.22 μm membrane filter. The organic layer was sent to Philippine Institute of Pure and Applied Chemistry (PIPAC) for GC-MS analysis. The composition of the sample was determined by injecting 1 μL of sample using Shimadzu GC-MS-QP2010 Ultra. The analytes were carried through DB-5 ms column (30 m, 0.25 mmID, 0.25 μm film thickness). The oven temperature program used was as follows: initially at 50°C held for 8 min; increased to 250°C at a rate of 10°C/min held for 10 min. The m/z range monitored was 30–800 with an event time of 0.30 s. The peaks were identified using the following criteria: highest match and > 80% similarity index in the NIST 08 library search. The mean % peak area of the replicates was used to compute the % relative abundance of each compound as follows (Imron et al., 2019):


%relativeabundance=

(%peakareaatday 0-%peakareaatday 14%peakareaatday 0)×100

### 2.5 Whole genome sequence analysis

#### 2.5.1 Whole genome sequencing

Isolate KLMG-HD-125 was grown on nutrient agar and sent to Macrogen, Inc., South Korea for whole genome sequencing by Illumina HiSeq X. TruSeq Nano DNA Library Prep Kit was used to generate high-quality DNA libraries.

#### 2.5.2 Quality assessment and trimming of raw reads

The quality of the Illumina raw reads was evaluated using FastQC v.0.12.1 (Andrews, 2010), which reported the total sequences, sequence length, and the %GC. Subsequently, trimming and filtering of raw reads were performed by FastP v0.24.1 (Chen et al., 2018), which also provided the mean length, total reads, Q20 bases, Q30 bases, and GC content before and after filtering.

#### 2.5.3 Assembly and annotation of draft genome

The assembly of the raw reads was performed using the default parameters of Unicycler v0.5.1 (Wick et al., 2017). The assembled draft genome was then polished using Pilon v1.18 (Walker et al., 2014). CheckM v1.1.0 (Parks et al., 2015) was used to check for contamination in the assembled draft genome, while BUSCO v5.8.3 (Manni et al., 2021) and BAKTA (Schwengers et al., 2021) were employed to check completeness and coverage of the assembly. PlasmidFinder (Carattoli et al., 2014) checked for the presence of plasmid. Quality Assessment Tool (QUAST) v5.3.0 (Gurevich et al., 2013) was used to determine the contiguity of the assembly. Genome-to-Genome Distance Calculator (GGDC) (Meier-Kolthoff et al., 2021) was utilized to estimate genomic relatedness by determining the digital DDH (dDDH) value. Prokka v1.14.5 (Seemann, 2014) was used to annotate the genes. To determine the genes in hydrocarbon metabolic pathways, the annotated draft genome was employed against the Kyoto Encyclopedia of Genes and Genomes (KEGG) database (Kanehisa et al., 2015). To identify the genes involved in hydrocarbon degradation and biosurfactant production, the genome was queried against the Hydrocarbon Aerobic Degradation Enzymes and Genes (HADEG) database (Rojas-Vargas et al., 2023) using ProteinOrtho v6.3.5 (Lechner et al., 2011). The presence of virulence genes was detected in Virulence Factor Database (VFDB) (Liu et al., 2021).

### 2.6 Orthology-based phylogenomic analysis

A phylogenomic analysis was performed on the assembled draft genome of isolate KLMG-HD-125 with the curated whole genome sequence of each type species of *Klebsiella* retrieved from the National Center for Biotechnology Information (NCBI) GenBank database (Benson et al., 2014) and *Escherichia coli* ATCC 11775 as the outgroup. The phylogenomic tree was generated using Orthofinder v3.1.0 (Emms and Kelly, 2019) based on 1,869 orthogroups with 100% of species carrying single-copy genes in any orthogroup. DIAMOND (double index alignment of next-generation sequencing data) (Buchfink et al., 2014) was used for sequence similarity searches, which were then aligned using FAMSA (Fast and accurate multiple sequence alignment of huge protein families) (Deorowicz et al., 2016). Interactive Tree Of Life (iTOL) v5 (Letunic and Bork, 2021) was used to visualize the tree.

### 2.7 Genome-based prediction of hydrocarbon biodegradation pathways

The function and identity of each gene associated with alkane and aromatic hydrocarbon degradation were determined through protein sequence similarity searches using the Basic Local Alignment Search Tool (BLAST) against the UniProt database (Ahmad et al., 2025). Predicted hydrocarbon biodegradation pathways were reconstructed based on annotations and curated references from the KEGG and HADEG databases and subsequently visualized using ChemDraw.

## 3 Results

### 3.1 Phenotypic characteristics

A total of 110 putative *Klebsiella* species were obtained from the microbial consortia. All purified isolates were Gram-negative, rod-shaped with single to short chain arrangements under 400X magnification. After 24 h on SCAI, all isolates formed slight to moderate abundance, yellow coloration, round, mucoid, and convex colonies. These results are consistent with Van Kregten et al. (1984) where *K. pneumoniae* and *K. oxytoca* appeared as yellow, dome-shaped, and often mucoidal colonies on SCAI.

### 3.2 Hydrocarbon degradation efficiencies

The 110 putative *Klebsiella* isolates from the microbial consortium exhibited varying hydrocarbon-degrading capabilities (Supplementary Figure 1). Eleven isolates demonstrated substantial degradation efficiency (≥66%) across all four hydrocarbons tested although the degradation periods varied (4–25 days) depending on both the isolate and hydrocarbon (Table 1). Hexadecane showed the highest average degradation (80.66%). Xylene generally requires the longest degradation periods. However, when degradation efficiency and period are considered, no single isolate is clearly superior among other isolates on any hydrocarbon. Taken together, the degradation efficiencies of the 11 isolates on all tested hydrocarbons significantly differed from each other, suggesting the uniqueness of each isolate. Isolate KLMG-HD-125 was subsequently chosen for analysis of diesel degradation and whole genome sequence due to its consistent degradation performance across multiple hydrocarbon substrates described as follows.

**TABLE 1:** 2,6-DCPIP assay of 11 *Klebsiella pneumoniae* isolates showing ≥ 66% degradation efficiency on all hydrocarbons.

Isolate	Diesel (%)	Period (days)	Hexadecane (%)	Period (days)	Hexane (%)	Period (days)	Xylene (%)	Period (days)
KLMG-HD-6	88.96	10	82.23	6	75.64	8	78.02	10
KLMG-HD-70	74.73	8	85.54	8	69.56	8	75.75	17
KLMG-HD-73	84.25	8	90.25	6	78.94	12	76.33	12
KLMG-HD-79	76.19	6	79.84	9	80.55	6	81.45	14
KLMG-HD-110	79.45	9	79.45	6	90.56	9	73.42	9
KLMG-HD-114	87.06	9	89.56	14	88.35	9	77.30	13
KLMG-HD-120	85.56	8	77.80	6	78.79	8	77.01	14
KLMG-HD-121	77.38	4	74.32	7	81.30	4	80.16	25
KLMG-HD-125	87.32	9	66.86	4	85.70	9	81.84	9
KLMG-HD-149	85.84	8	93.06	6	77.56	12	73.51	12
KLMG-HD-152	78.21	4	68.33	12	71.43	16	77.26	14

### 3.3 Biochemical traits

The 11 isolates with ≥ 66% degradation efficiency on the tested hydrocarbons displayed positive reactions for catalase, urease, and Voges-Proskauer tests, citrate utilization, indole and H_2_S production, and fermentation of glucose, lactose, and fructose, while testing negative for oxidase and methyl red. These characteristics were consistent with *Klebsiella* presented in Bergey’s manual of systematic bacteriology (Brenner et al., 2005).

### 3.4 Diesel degradation

**FIGURE 1:**
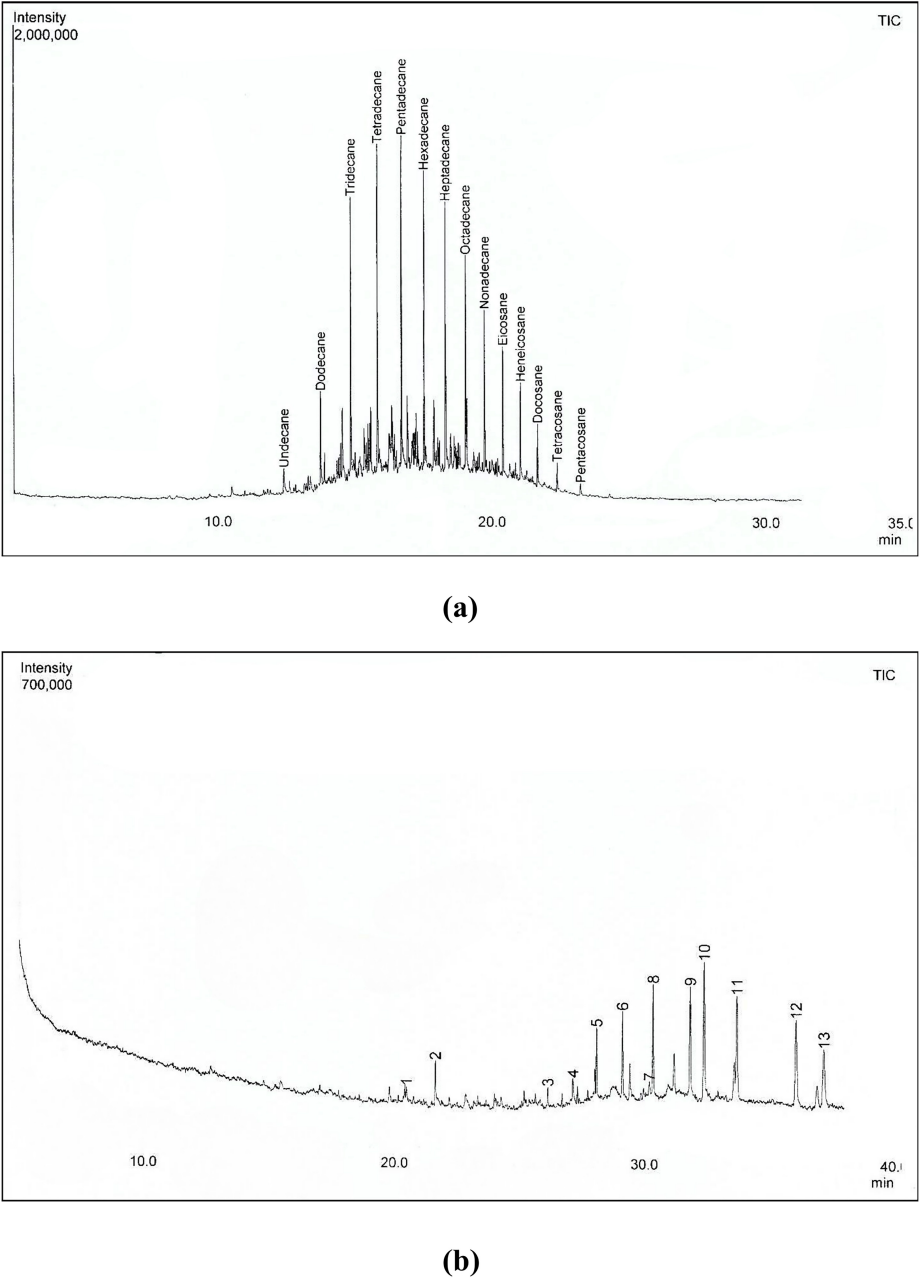
GC-MS chromatograms of **(a)** control [diesel alone] and **(b)** diesel after 14 days of biodegradation by KLMG-HD-125. (1) Phenol, 2,4-bis(1,1-dimethylethyl)-; (2) Eicosane; (3) Pentadecane,8-hexyl-; (4) Tetratetracontane; (5) Tetracosane; (6) Hexatriacontane; (7) Hexanedioic acid, bis(2-ethylhexyl) ester; (8) Tetratriacontane; (9) Tritriacontane; (10) 1,2-benzenedicarboxylic acid, mono(2-ethylhexyl) ester; (11) Triacontane; (12) Pentatriacontane; (13) Terephthalic acid, di(2-ethylhexyl) ester.

Figure 1a and Table 2 show that the diesel used in this study was largely composed of straight-chain aliphatics ranging from C11 to C25. The absence of polyaromatic hydrocarbons may be attributed to the cell-free extraction process as *n*-hexane is not efficient in extracting aromatic hydrocarbons. The *n*-hexane cannot engage in π–π interactions with aromatics, hence it cannot form stabilizing interactions with the aromatic rings (Von Lau et al., 2014).

**TABLE 2:** GC-MS analysis of products of diesel degradation by *Klebsiella pneumoniae* (KLMG-HD-125).

Name of compounds	Chemical formula	Carbon chain length	Compound type	Retention time (min)	% Relative abundance (day 0)	% Relative abundance (day 14)	Degradation status
Undecane	C_11_H_24_	11	Straight-chain aliphatic	12.386	1.195	0	Complete
Dodecane	C_12_H_26_	12	Straight-chain aliphatic	13.725	4.905	0	Complete
Tridecane	C_13_H_28_	13	Straight-chain aliphatic	14.825	11.945	0	Complete
Tetradecane	C_14_H_30_	14	Straight-chain aliphatic	15.796	14.6	0	Complete
Pentadecane	C_15_H_32_	15	Straight-chain aliphatic	16.684	13.355	0	Complete
Hexadecane	C_16_H_34_	16	Straight-chain aliphatic	17.512	12.13	0	Complete
Heptadecane	C_17_H_36_	17	Straight-chain aliphatic	18.292	13.14	0	Complete
Octadecane	C_18_H_38_	18	Straight-chain aliphatic	19.03	7.89	0	Complete
Nonadecane	C_19_H_40_	19	Straight-chain aliphatic	19.732	6.76	0	Complete
Eicosane	C_20_H_42_	20	Straight-chain aliphatic	20.401	5.36	2.845	Partial (−46.92%)
Heneicosane	C_21_H_44_	21	Straight-chain aliphatic	21.04	3.92	0	Complete
Docosane	C_22_H_46_	22	Straight-chain aliphatic	21.671	2.605	0	Complete
Tetracosane	C_24_H_50_	24	Straight-chain aliphatic	22.387	1.475	4.125	Accumulated (+179.67%)
Pentacosane	C_25_H_52_	25	Straight-chain aliphatic	23.246	0.725	0	Complete
Phenol, 2,4-bis(1,1-dimethylethyl)-	C_17_H_30_OSi	17	Phenolic	20.4825	0	1.475	Formed
8-Hexylpentadecane	C_21_H_44_	21	Branched-chain aliphatic	26.145	0	1.335	Formed
Tetratetracontane	C_44_H_90_	44	Straight-chain aliphatic	27.1415	0	2.6	Formed
Hexatriacontane	C_36_H_74_	36	Straight-chain aliphatic	29.124	0	6.765	Formed
Hexanedioic acid, bis(2-ethylhexyl) ester	C_22_H_42_O_4_	22	Ester	30.1945	0	1.535	Formed
Tetratriacontane	C_34_H_70_	34	Straight-chain aliphatic	30.3355	0	9.48	Formed
Tritriacontane	C_33_H_68_	33	Straight-chain aliphatic	31.8185	0	12.425	Formed
1,2-Benzenedicarboxylic acid, mono(2-ethylhexyl) ester	C_16_H_22_O_4_	16	Ester	32.3685	0	16.615	Formed
Triacontane	C_30_H_62_	30	Straight-chain aliphatic	33.6755	0	14.125	Formed
Pentatriacontane	C_35_H_72_	35	Straight-chain aliphatic	36.049	0	15.67	Formed
Di-(2-ethylhexyl) phthalate	C_24_H_38_O_4_	24	Phthalate	37.1625	0	11	Formed

After 14 days of incubation, the GC-MS profile demonstrates complete degradation of short- and mid-chain n-alkanes (C11–C25) by KLMG-HD-125, except eicosane (C20) and tetracosane (C24) (Figure 1b and Table 2). Although eicosane was detected after 14 days, its abundance decreased by 2.515%, suggesting partial degradation. In contrast, the abundance of tetracosane increased by 2.65% (Table 2), which is likely associated with complete degradation of longer chained aliphatic, pentacosane (C25), contributing to relative enrichment of tetracosane. Oxygenated metabolites such as phenolic compounds, esters and acids were detected after 14 days as well as the long-chain and complex hydrocarbons, including triacontane (C30), tritriacontane (C33), pentatriacontane (C35), hexatriacontane (C36), and tetratetracontane (C44).

### 3.5 Whole genome sequencing and genomic characterization

#### 3.5.1 Quality of raw reads

The raw Illumina read generated a total of 2.78 Gb from 18.4 million paired-end reads. Raw sequencing data showed 57.20% GC content with Q20 and Q30 scores of 97.1% and 92.6%, respectively (Table 3). After FastP processing, the input file showed little adapter trimming (∼1.074% for read 1 and read 2), indicating that the raw reads had already been trimmed. After filtering, only 0.31 and 0.39% increase in the Q20 and Q30, respectively, were seen, confirming the high quality of the raw sequencing data.

**TABLE 3:** Quality of reads before and after using Fastp.

Stage	Total reads	Total bases	Q20 bases	Q30 bases	GC content
Before filtering	18.40 M	2.78 Gb	2.70 G (97.12%)	2.57 G (92.56%)	57.20%
After filtering	18.20 M	2.75 Gb	2.68 G (97.43%)	2.55 G (92.95%)	57.20%

#### 3.5.2 Genome assembly quality and annotation

Assessment of the genome assembly with QUAST produced 73 contigs with no mismatches (Table 4). BUSCO analysis showed 98.4% completeness, 97.46% single-copy, 0.8% duplicated, 0% fragmented, and 1.6% missing genes out of 124 BUSCOs, indicating a high-quality assembly. The draft genome contains 5,138 coding sequences, six rRNA genes, one repeat region, 77 tRNAs, and one tmRNA. It also harbors an IncF-type plasmid that contains *IncFIA*(HI1) and *IncFIB*(K) replicon genes, which are common in *Enterobacteriaceae*, especially *E. coli* and *Klebsiella* species (Zhang et al., 2023). This plasmid carries antimicrobial resistance genes but not genes associated with hydrocarbon metabolism.

**TABLE 4:** Summary statistics of draft genome of KLMG-HD-125.

Genomic data	Isolate KLMG-HD-125
Raw reads	
Total reads^1^	18.40 M
Total bases^1^	2.78 Gb
Mean length^1^	151 bp
Quality controlled reads	
Total sequences^1^	18.20 M
Total bases^1^	2.75 Gb
Mean length^1^	150 bp
Percent recovery^1^	98.93%
Percent GC (%)^1^	57.20%
Assembled draft genome	
Completeness (%)^2^	100
Contamination (%)^2^	0.39
Coverage (X)	250
Total length^3^	5.55 Mb
Number of contigs^3^	73
Largest contig^2^	0.90 Mb
Genes^4^	5,220
CDS^5^	5,138
tRNA^5^	77
rRNA^5^	6
tmRNA^5^	1
N50^6^	314.52 kb
N90^6^	86.23 kb
N_count^6^	0

^1^Assessed using FastQC.

^2^Assessed using CheckM.

^3^Assessed using BUSCO.

^4^Assessed using BAKTA.

^5^Assessed using Prokka.

^6^Assessed using QUAST.

#### 3.5.3 Taxonomic identity of KLMG-HD-125

**FIGURE 2:**
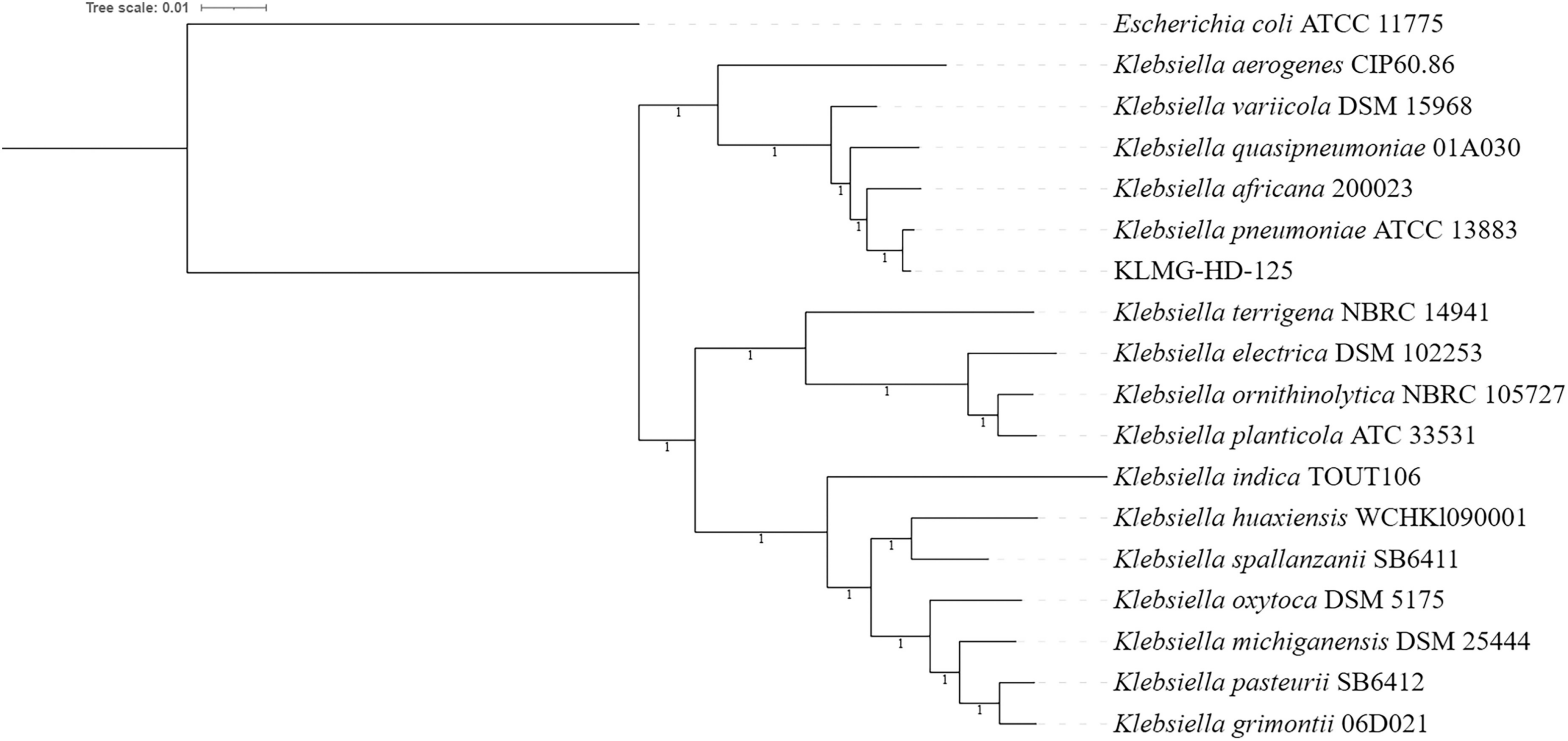
Phylogenomic tree based on OrthoFinder analysis using 1,869 orthogroups. Branch values correspond to OrthoFinder support values.

Phylogenomic analysis using 1,869 single-copy orthogroups confirmed that isolate KLMG-HD-125 belongs to *Klebsiella pneumoniae*, clustering with reference strain *K. pneumoniae* ATCC 13883 (Figure 2). The ANI value (98.96%) and dDDH value (93.20%) between the isolate and *Klebsiella pneumoniae* ATCC 13883 strongly support the species identity of KLMG-HD-125.

#### 3.5.4 Alkane degradation genes

**FIGURE 3:**
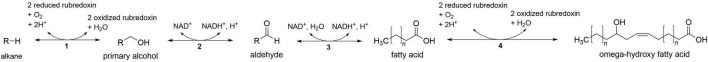
Proposed alkane degradation pathway of isolate KLMG-HD-125. (1) Alkane monooxygenase (*ladA*); (2) primary alcohol dehydrogenase (*adhP*); (3) aldehyde dehydrogenase (*adh*); (4) alkane monooxygenase (*ladA*).

The genome of KLMG-HD-125 carries genes attributed to alkane degradation, namely, *ladA* (FMN-dependent monooxygenase), *adhP* (primary alcohol dehydrogenase), and *adh* (aldehyde-alcohol dehydrogenase, long-chain aldehyde dehydrogenase), which support the general alkane pathway in Figure 3. These ω-hydroxy acids can enter the tricarboxylic acid (TCA) cycle via β-oxidation pathway (Li et al., 2008).

Other genes such as *ssuD*, *ahpF, ahpC*, *blc*, and *fadL*, which do not directly function in alkane degradation but support this process, are also found in the genome of KLMG-HD-125. Alkanesulfonate monooxygenase (*ssuD*) is a broad range FMNH(2)-dependent monooxygenase catalyzing the oxygenolytic conversion of alkanesulfonates to sulfite and the corresponding aldehydes (Eichhorn et al., 1999). AhpCF peroxidase system (*ahpCF*) contributes to decreasing toxic peroxides produced during alkane degradation (Rocha and Smith, 1999). Blc (*blc*) is a membrane-associated lipoprotein that protects the cell against membrane stress from increased fatty acids concentration during alkane oxidation (Campanacci et al., 2004). FadL (*fadL*) is a long-chain fatty acid outer-membrane transporter that transports long-chain fatty acids (and possibly alkanes) across outer membrane (Black and DiRusso, 2003).

#### 3.5.5 Xylene degradation genes

**FIGURE 4:**
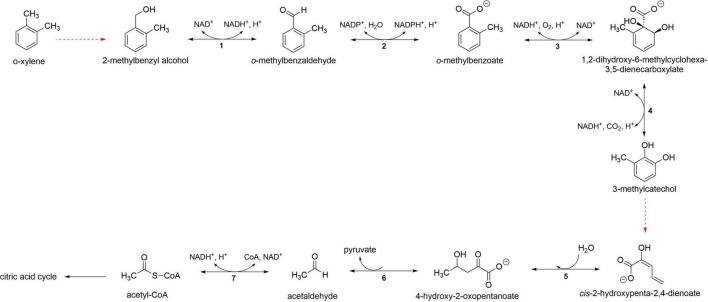
Proposed xylene degradation pathway of isolate KLMG-HD-125. (1) Aryl-alcohol dehydrogenase (*xylB*); (2) NAD-dependent aldehyde dehydrogenase (*xylC*); (3) benzoate/toluate 1,2-dioxygenase reductase component (*benC*); (4) 1,2-dihydroxycyclohexa-3,5-diene-1-carboxylate dehydrogenase (*benD*); (5) 2-keto-4-pentenoate hydratase (*mhpD*); (6) 4-hydroxy 2-oxovalerate aldolase (*mhpE*); (7) acetaldehyde dehydrogenase (*mhpF*).

KLMG-HD-125 harbors genes that encode enzymes catalyzing several steps in xylene degradation, including *xylB* (aryl-alcohol dehydrogenase), *xylC* (NAD-dependent aldehyde dehydrogenase), *benC* (benzoate/toluate 1,2-dioxygenase), *benD* (1,2-dihydroxycyclohexa-3,5-diene-1-carboxylate dehydrogenase), *mhpD* (2-keto-4-pentenoate hydratase), *mhpE* (4-hydroxy 2-oxovalerate aldolase) and *mhpF* (acetaldehyde dehydrogenase) (Figure 4). However, the gene required to convert methylcatechol to cis,2-hydroxypenta-2,4-dienoate was not found when the genome of KLMG-HD-125 was queried in KEGG and HADEG databases, which implies the use of alternative, non-canonical enzymes.

#### 3.5.6 Genes for biofilm production

Major factors that affect biofilm formation of *K. pneumoniae* include capsule, fimbriae, quorum sensing, and nutritional conditions (Li and Ni, 2023). KLMG-HD-125 contains *wzb* and *wzc*, which encode protein tyrosine kinase for the synthesis and regulation of bacterial capsular polysaccharide (CPS). *treC* (trehalose-6-phosphate hydrolase), which modulates the production of polysaccharide capsule, is also present (Wu et al., 2011).

In *K. pneumoniae*, biofilm formation is mainly mediated by Type 3 fimbriae (Allen et al., 1991), encoded by *mrkABCDF*. KLMG-HD-125 produces MrkD (*mrkD)*, which displays adhesion characteristics of appendages and determines specificity of fimbriae binding (Murphy and Clegg, 2012). MrkD also contributes to form dense *K. pneumoniae* biofilms (Ashwath et al., 2022). Of the *fimAICDFGHK* that expresses Type 1 fimbriae, KLMG-HD-125 harbors *fimC*, *fimF*, *fimG*, and *fimH*. KLMG-HD-125 also possesses *ecpRABCDE* operon, which expresses *Escherichia coli* common pilus (ECP), a fimbrial adhesin involved in adhesion, early biofilm formation, and host colonization (Alcántar-Curiel et al., 2013), and which is also considered a virulence determinant in *Enterobacteriaceae* (Munhoz et al., 2023). KLMG-HD-125 also carries *pgaA*, *pgaB*, and *pgaC* of the operon *pgaABCD*, which codes for the enzymes and machinery to synthesize and export poly-N-acetylglucosamine (Cywes-Bentley et al., 2013), a crucial polysaccharide that forms the backbone of the bacterial biofilm matrix, for attachment, protection from immune cells/antibiotics, and persistence.

KLMG-HD-125 carries *sdiA* (SdiA) and *luxS* (LuxS). By sensing external signals (acyl-homoserine lactones), SdiA influences bacterial communication and behavior, impacting biofilm formation and pathogenicity (Pacheco et al., 2021). LuxS is essential for the synthesis of the quorum-sensing signal autoinducer-2 (AI-2). KLMG-HD-125 also harbors *tqsA* and the *lsrCD* system, which, respectively, encode a protein involved in the transport of AI-2 and an ABC transporter responsible for AI-2 import.

#### 3.5.7 Oxidative stress response genes

KLMG-HD-125 contains several genes that code for superoxide dismutases (*sodA*, *sodB*), catalases (*katG*, *katE*), peroxidases (*ahpC*, *bcp*), and other genes in response to oxidative stress. KLMG-HD-125 also contains the OxyR and SoxRS systems, which are the major oxidative-stress regulatory systems in *Klebsiella pneumoniae* (Chiang and Schellhorn, 2012).

#### 3.5.8 Membrane and solvent stress resistance genes

The KLMG-HD-125 genome also features *acrA*, *acrB*, *tolC, ompF*, *ompC*, *dnaK, groE*, *groL*, and *hsp20*. The *acrAB* gene cluster encodes the AcrAB-TolC efflux pump, which pumps out toxic compounds (Bhogal et al., 2025). OmpF and OmpC are outer membrane proteins that can physically control membrane permeability during hydrocarbon exposure. The stress-response chaperones, including DnaK, GroEL, and Hsp20, are known to be induced during organic solvent and membrane stress to protect against protein denaturation caused by hydrocarbon exposure (Fayet et al., 1989; Khaskheli et al., 2015).

#### 3.5.9 Virulence genes

KLMG-HD-125 carries 11 virulence genes, including *entA*, *entB*, and *fepC* (iron acquisition) and *ompA* (outer membrane protein). However, the hallmark hypervirulent genes such as *rmpA, iucA*, *iroB*, and *peg-344* (Hetta et al., 2025) were not found in KLMG-HD-125. These genes are often found on a large virulence plasmid, which KLMG-HD-125 does not carry.

## 4 Discussion

Eleven putative *Klebsiella* isolates from the enriched microbial consortium from Guimaras displayed significantly different hydrocarbon degradation efficiencies of at least 66%, indicating diversity at the species or strain level. When compared with the parent consortium incubated for 7 days at 30°C (Rodriguez et al., 2023), some isolates performed better while others were worse depending on the substrate and incubation period. For example, isolates KLMG-HD-121 (81.30% on hexane after 4 days) and KLMG-HD-149 (93.06% on hexadecane after 6 days) outperformed the consortium. Some isolates produced higher diesel degradation values than the consortia but at longer incubation period of up to 10 days. Each isolate showed higher values for xylene but at extended incubation period. These results suggest that particular putative *Klebsiella* isolate may be potentially employed in rehabilitation of specific type of hydrocarbon. However, while 2,6-DCPIP provides degradation potential, the values represent enzymatic activity rather than quantitative hydrocarbon removal and must be validated through direct analytical methods such as gas chromatography for definitive degradation rates.

Whole genome sequencing and phylogenomic analysis established that KLMG-HD-125 belongs to *Klebsiella pneumoniae*. The genome size, GC content, and gene count are consistent with previously sequenced *K. pneumoniae* strains (Holt et al., 2015), supporting its taxonomic placement.

GC-MS analysis indicated that isolate KLMG-HD-125 prefers C11–C25 n-alkanes except eicosane (C20), which was only partially degraded. The emergence of oxygenated metabolites such as phenolic compounds, esters and acids after growth on diesel suggests oxidative biodegradation pathways. The detection of long-chain and complex hydrocarbons, including triacontane (C30), tritriacontane (C33), pentatriacontane (C35), hexatriacontane (C36), and tetratetracontane (C44), may be attributed to transformation products, residual recalcitrant diesel fractions, or co-eluting unresolved complex mixtures (UCM). Similar results were obtained on GC-MS analysis of petroleum degradation by *K. pneumoniae* ATCC13883, where C11–C20 petroleum components were nearly completely degraded after 7 days at 25°C (Ozyurek and Bilkay, 2018). However, in contrast to KLMG-HD-125, *K. pneumoniae* ATCC13883 degraded C20 at 93%. *K. pneumoniae* isolated from a petroleum refinery in Nanjing, China degraded > 80% of C12 within 6 days, whereas degradation of longer-chain alkanes (C16–C24) was at below 50%, as determined by measuring residual hydrocarbon concentration via A_256_ (You et al., 2018). Result deviations among the isolates may be due to strain-specific variation in hydrocarbon metabolism among *K. pneumoniae* or to different assay conditions.

Phenotypic analyses clearly demonstrated that KLMG-HD-125 can degrade small- to medium-length n-alkanes. However, the canonical alkane hydroxylases such as *alkB* (Williams and Austin, 2022) or *CYP153* (Bernhardt, 2006) were absent in its genome. Instead, KLMG-HD-125 harbors *ladA* that codes for LadA-like FMN-dependent monooxygenase (NtaA/SnaA/SoxA family), which has been structurally and functionally characterized as long-chain alkane hydroxylases that catalyze the terminal oxidation of n-alkanes to primary alcohols (Li et al., 2008). *ladA* was likewise identified in hydrocarbon-degrading metagenomes associated with terminal oxidation pathways (Perera et al., 2022). Subsequent conversion steps in KLMG-HD-125 are supported by alcohol and aldehyde dehydrogenases (*adhP* and *adh*), leading to the fatty acid production that can enter the β-oxidation pathway (Fathepure, 2014). Together, LadA, primary alcohol dehydrogenase (*adhP*) and long-chain aldehyde dehydrogenase (*adh*) suggest terminal oxidation of alkane (Ji et al., 2013). However, GC-MS analysis did not detect products of terminal oxidation such as fatty acids, ω-hydroxy fatty acids and dicarboxylic acids nor secondary alcohols/ketones from subterminal oxidation, likely due to these products being channeled to the central metabolic pathways or due to the extraction and GC-MS detection limits. The absence of canonical xylene monooxygenase and methylcatechol meta-cleavage genes in the genome, despite significant xylene degradation as measured by 2,6-DCPIP assay, suggests that KLMG-HD-125 may rely on broad-substrate oxygenases, incomplete pathways, or cometabolic processes rather than a complete archetype xylene degradation pathway. These are not surprising since *K. pneumoniae* is a generalist heterotroph rather than an obligate hydrocarbon degrader (Blin et al., 2017). It is also possible that the Illumina short read assembly could have missed these genes.

The presence of biofilm-related genes in the genome of KLMG-HD-125 suggests niche persistence, stabilization of dispersed oil droplets by forming protective communities at the oil–water interface, and biosurfactant production (Omarova et al., 2019). However, KLMG-HD-125 did not produce biofilm nor biosurfactant when tested in the laboratory by following the methods of Christensen et al. (1985) and Nwaguma et al. (2016), suggesting sub-optimal environmental conditions during growth and assay (Ribeiro et al., 2023).

KLMG-HD-125 is equipped with genetic features that provide stress adaptation, including against ROS that may be inadvertently produced during aromatic or long-chain hydrocarbon degradation (Maslovska et al., 2023) by expressing ROS-detoxifying enzymes such as superoxide dismutases, catalases and peroxidases. The presence of AcrAB–TolC efflux system, outer membrane proteins, and molecular chaperones can confer protection to KLMG-HD-125 against toxic hydrocarbon accumulation, membrane permeability disruption, and protein denaturation, respectively.

The presence of virulence factors such as siderophore-mediated iron acquisition systems, ECP and OmpA indicates that KLMG-HD-125 possesses multiple strategies for host colonization, nutrient acquisition, and resistance to host immune defense. However, many commensal and environmental *Enterobacteriaceae* harbor similar virulence-associated genes without causing disease (Holt et al., 2015). Given its source, KLMG-HD-125 is likely an opportunistic rather than an obligate human pathogen.

This study demonstrates that KLMG-HD-125 possesses phenotypic and genomic attributes supporting hydrocarbon remediation, specifically of n-alkanes, as well as ecological fitness for persistence in petroleum-impacted environments. However, its potential pathogenicity warrants careful risk evaluation prior to environmental application.

Because the hydrocarbon degradation pathways described here are inferred largely from genomic evidence, additional experimental studies will be necessary to verify these predicted metabolic functions. In particular, incorporating transcriptomic and proteomic approaches would further reveal how genes are expressed and how specific pathways are activated during hydrocarbon degradation. Targeted RT-qPCR assays could be used to monitor the transcriptional responses of *ladA*, *adhP*, *adh*, and key stress-response genes when cells are exposed to high hydrocarbon concentrations. Broader transcriptomic surveys would also allow identification of a wider set of hydrocarbon-responsive genes that may not be evident from genome data alone. The putative virulence-associated genes detected in the genome warrant further examination to determine whether they are expressed and contribute to any measurable phenotypes in this strain. Complementary proteomic analyses would then provide direct evidence for the production and relative amounts of enzymes involved in alkane oxidation, β-oxidation, oxidative-stress processes, and potentially any virulence-related functions. Together, these evidences would offer a more complete picture of the metabolic activity underlying hydrocarbon degradation and provide a stronger basis for evaluating the bioremediation potential and ecological behavior of KLMG-HD-125.

## Data Availability

The whole genome sequence project has been deposited at DDBJ/ENA/GenBank under the accession JBSOLS010000000.
