# Supplementary material for: Hydrocarbon degradation and genomic insights of Klebsiella pneumoniae from oil-contaminated soils in Guimaras Island, Philippines
Source: Front Microbiol. 2026 Mar 18;17:1782430. doi: 10.3389/fmicb.2026.1782430 (PMC13040789; doi:10.3389/fmicb.2026.1782430)
Supplement: Supplementary file 1 [file Table_1.docx]

Supplementary Material

# Supplementary Figure


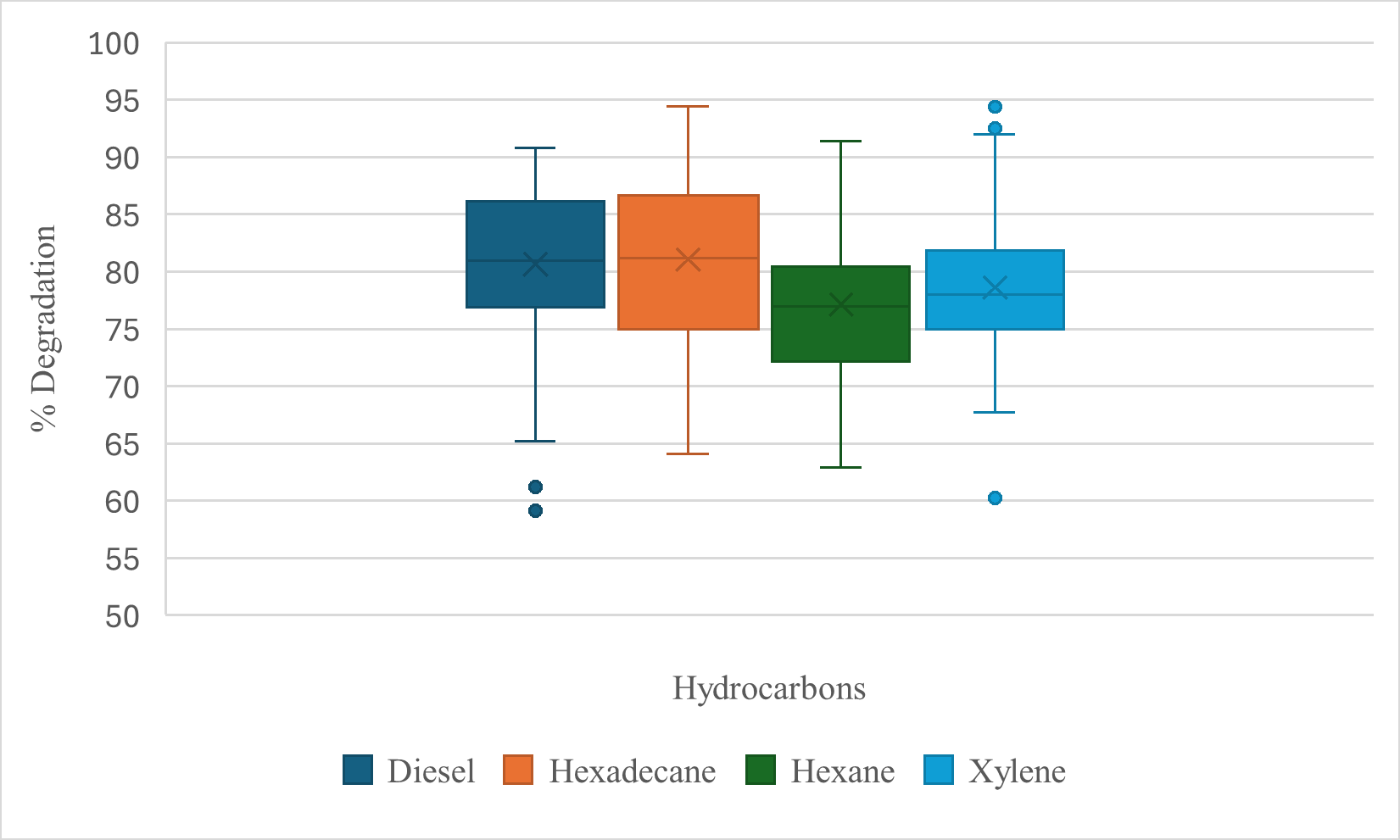


**Supplementary Figure 1.** Box plot showing the hydrocarbon-degrading capabilities of 110 putative Klebsiella isolates from diesel-enriched microbial consortium as measured by reduction of 2,6-dichlorophenolindophenol (DCPIP).
